# The impact of early integrated palliative care on symptom burden and quality of life in patients with advanced breast cancer

**DOI:** 10.3389/fonc.2026.1817750

**Published:** 2026-05-13

**Authors:** Shiying Li, Weina Wang, Chengxin Bai, Jianping Wang

**Affiliations:** 1 The First Hospital of Hebei Medical University, Shijiazhuang, China; 2 Breast Center, the Fourth Hospital of Hebei Medical University, Shijiazhuang, China

**Keywords:** advanced breast cancer, early integrated palliative care, end-of-life chemotherapy, psychosocial outcomes, quality of life, symptom burden

## Abstract

Patients with advanced breast cancer endure a high symptom burden and impaired quality of life. This study evaluated the impact of Early Integrated Palliative Care (EIPC) on these outcomes. Methods: A retrospective cohort study analyzed patients with advanced breast cancer treated between November 2018 and December 2021. Patients were categorized into an EIPC group or a usual care group based on the care model received. Symptom burden (SDS) and sleep quality (PSQI) were assessed at baseline, 6, 12, 18, and 24 weeks. Quality of life (FACT-B), hope (HHI), and anxiety/depression (HADS) were evaluated at baseline and 24 weeks. End-of-life chemotherapy rates were recorded. Results: Of 200 included patients, 88 received EIPC, and 112 received usual care. The EIPC group demonstrated significantly lower SDS scores from week 6 onward (24 weeks: 27.57 vs. 29.71, P = 0.002) and lower PSQI scores (24 weeks: 7.21 vs. 8.19, P = 0.002). At 24 weeks, the EIPC group had significantly higher FACT-B scores in physical, social/family, emotional, and functional well-being domains (all P<0.05), higher HHI scores (positive attitude: 13.06 vs. 12.44, P = 0.004), and lower HADS depression (6.45 vs. 7.36, P = 0.002) and anxiety scores (6.23 vs. 7.18, P = 0.001). The rate of chemotherapy in the last 6 weeks of life was significantly lower in the EIPC group (20.45% vs. 40.18%, P = 0.003). Conclusion: EIPC alleviates symptom burden, improves quality of life, psychological well-being, and sleep, and promotes more goal-concordant end-of-life care in patients with advanced breast cancer.

## 1 Introduction

Advanced breast cancer (ABC), including metastatic (stage IV) and locally advanced, inoperable disease, is an important and evolving frontier in oncology (1). Patients diagnosed with ABC face profound and multifaceted burdens characterized not only by disease progression but also by a range of severe symptom burdens, including pain, debilitating fatigue, emotional distress, and functional impairment (2, 3). These factors collectively have a devastating impact on their quality of life (QoL) (4). Although advances in targeted therapy and immunotherapy have modestly extended survival for certain subtypes, the primary clinical paradigm remains highly focused on tumor control (5, 6). This often inadvertently leads to fragmented care, where management of physical and psychological suffering is passively addressed or deferred until the final stages (7, 8). Consequently, there is a critical gap between life-prolonging treatments and patient-centered holistic support throughout the course of the disease.

This gap has catalyzed a paradigm shift towards integrated care models. The biopsychosocial model underscores the indivisibility of physical symptoms from psychological and social well-being (8, 9). Research on other solid tumors, particularly lung cancer, provides compelling evidence that integrating specialized palliative care early with standard oncologic treatment can improve patient-reported outcomes, mood, and even survival (10). The proposed mechanisms are multifactorial. Active symptom management alleviates physical suffering, structured psychosocial interventions address anxiety and depression, and early and ongoing communication about prognosis and values through advance care planning (ACP) fosters realistic expectations and aligns medical interventions with patient goals (11, 12). These components work synergistically to enhance coping and reduce suffering from the time of diagnosis.

However, the evidence base for advanced cancer, particularly within specific healthcare settings, requires further validation. Therefore, this study aims to evaluate the comprehensive impact of structured Early Integrated Palliative Care (EIPC) intervention compared to usual oncologic care on patients with ABC. We conducted a retrospective cohort analysis assessing a broad range of outcomes. The novelty of this study lies in its detailed evaluation of a multidisciplinary EIPC model in this population, incorporating both subjective patient-reported metrics and objective clinical behavior metrics. By providing empirical data, this study seeks to strengthen the argument for standardizing EIPC as an essential component of high-quality, comprehensive care for patients with advanced cancer. Importantly, EIPC should not be interpreted as an alternative to active oncologic treatment but rather as a complementary approach within a “simultaneous care” model. In this framework, palliative care is delivered alongside disease-directed therapies, including chemotherapy, with the aim of optimizing symptom control, supporting decision-making, and aligning treatments with patient goals. Evidence suggests that such integration does not reduce appropriate treatment but instead reduces non-beneficial aggressive interventions near the end of life, including unnecessary chemotherapy, by facilitating timely communication and advance care planning.

## 2 Materials and methods

### 2.1 Study design and criteria

A retrospective analysis was conducted on 200 patients with advanced breast cancer admitted to our hospital from November 2018 to December 2021. Inclusion criteria were: (1) female patients aged ≥18 years; (2) histopathologically confirmed stage IV (metastatic) breast cancer (13); (3) Eastern Cooperative Oncology Group (ECOG) performance status score of 0-2; (4) expected survival period ≥12 months; (5) complete medical records and questionnaire survey data. Exclusion criteria included: (1) presence of severe communication barriers or cognitive dysfunction that prevented completion of the questionnaire assessment; (2) active, uncontrolled psychiatric disorders; (3) history of other malignancies; and (4) previous receipt of palliative care interventions.

Depending on the care model received, the 200 patients were defined into the usual care group (n=112) and the Early Integrated Palliative Care (EIPC) group (n=88). The usual care group was defined as receiving only routine supportive care from the oncology department without specific palliative care; the EIPC group was defined as receiving concurrent EIPC in addition to routine oncological care.

### 2.2 Ethical statement

This study protocol has been reviewed and approved by the Medical Ethics Committee of the Fourth Hospital of Hebei Medical University. The research was conducted in strict accordance with the Declaration of Helsinki and relevant medical research ethics norms. All patient data were anonymized (removing personal identifiers such as name, ID number, and contact information) to protect patient privacy. Due to the retrospective nature of the study, the medical records and questionnaire data used were existing clinical data, and the Ethics Committee approved the waiver of informed consent. Before data collection, the researchers signed a data confidentiality agreement to ensure that the research data would only be used for this study and not disclosed to third parties.

### 2.3 Care models

#### 2.3.1 Usual care

(1) Routine Health Education:​ Providing information on disease pathogenesis, treatment principles, and general self-management advice; (2) Basic Symptom Management:​ Administering medications (e.g., analgesics, antiemetics) as prescribed by the oncologist to manage treatment-related side effects; (3) General Supportive Care:​ Offering basic guidance on diet, sleep hygiene, and activity, encouraging patients to maintain a balanced diet and regular sleep schedule; (4) Monitoring:​ Regular assessment of vital signs and management of common complications, such as preventing pressure ulcers and respiratory infections through standard nursing practices.

#### 2.3.2 Early integrated palliative care

Within four weeks of diagnosis of advanced breast cancer, a structured, proactive, and multidisciplinary EIPC was initiated by a specialized palliative care team. (1) Multidisciplinary Team (MDT) Formation and Comprehensive Assessment: A dedicated MDT was established, comprising palliative care physicians, certified palliative care nurses, psychologists, dietitians, rehabilitation therapists, and social workers. At the initiation of EIPC, this team conducted a comprehensive baseline assessment using standardized scales for each patient to evaluate symptom burden, psychological distress, social support needs, nutritional status, and spiritual concerns. (2) Personalized Care Plan: Based on the comprehensive assessment, the MDT collaboratively developed a personalized EIPC care plan for each patient. This plan was discussed with the patient and their family to ensure alignment with their goals, values, and preferences; (3) Symptom-Targeted Management: Targeted interventions guided by regular Symptom Distress Scale (SDS) assessments addressed core symptoms such as nausea, vomiting, pain, dyspnea, and fatigue; (4) Structured Psychosocial Support: Psychologists provided individual Cognitive Behavioral Therapy (CBT) and Mindfulness-Based Stress Reduction (MBSR) sessions (45–60 minutes per month) to address anxiety and depression identified through the Hospital Anxiety and Depression Scale (HADS). Additionally, patient support group meetings were held every 8 weeks, facilitated by a psychologist and a palliative care nurse, to encourage peer support and sharing of coping strategies; (5) Communication and Advance Care Planning (ACP): The palliative care team facilitated ongoing discussions about disease understanding, prognosis, and treatment goals. They assisted patients in clarifying preferences for future care, including end-of-life decisions, and documented these discussions in the medical record; (6) Care Coordination and Practical Support: A social worker addressed practical needs such as financial issues and transportation, and connected patients with community resources. Dietitians and rehabilitation therapists provided tailored interventions to maintain nutritional status and physical function; (7) Integration and Follow-Up: The palliative care MDT regularly communicated with the primary oncology team through shared electronic health records and weekly interdisciplinary meetings. Follow-up consultations with a palliative care nurse or physician were scheduled at least monthly, with additional contacts as needed based on the patient’s condition.

### 2.4 Observation indicators

#### 2.4.1 Primary indicators

##### 2.4.1.1 Symptom distress scale

The SDS was used to assess the level of discomfort experienced by patients due to breast cancer-related symptoms before care and at 6, 12, 18, and 24 weeks after the initiation of care. This scale includes 13 items: nausea, vomiting, poor appetite, fatigue, taste changes, shortness of breath, pain, difficulty concentrating, sadness, appearance changes, worry, and functional limitations. It uses a Likert 5-point scale, where 1 indicates “no distress, ” and 5 indicates “extreme distress, ” with total scores ranging from 13 to 65. Higher total scores indicate more severe overall symptom distress. The Cronbach’s α coefficient for the Chinese version of the SDS is 0.85 (14).

##### 2.4.1.2 Functional assessment of cancer therapy-breast

The FACT-B was used to assess the quality of life of patients before care and 24 weeks after the initiation of care. This scale comprises 36 items across five dimensions: Physical Well-Being (PWB) (7 items), Social/Family Well-Being (SWB) (7 items), Emotional Well-Being (EWB) (6 items), Functional Well-Being (FWB) (7 items), and a breast cancer-specific subscale (BCS) (9 items). It uses a Likert 5-point scale, where 1 indicates “not at all” and 5 indicates “very much, ” with total scores ranging from 36 to 180. Higher scores indicate better quality of life and better conditions in each dimension. The Chinese version of FACT-B retains all items from the original questionnaire, with Cronbach’s α coefficients for the five dimensions ranging from 0.61 to 0.84 (15).

#### 2.4.2 Secondary indicators

##### 2.4.1.1 Herth hope index

The HHI was used to assess the level of hope in patients before care and 24 weeks after the initiation of care. This scale includes three dimensions: a positive attitude toward reality and the future, taking positive action, and maintaining close relationships with others. It consists of 12 items rated on a Likert 4-point scale, where 1 indicates “strongly disagree, ” and 4 indicates “strongly agree, ” with total scores ranging from 12 to 48. Higher scores indicate higher levels of hope. The Cronbach’s α coefficient for the Chinese version of the HHI is 0.85 (16).

##### 2.4.2.2 Hospital anxiety and depression scale

The HADS was used to assess the anxiety and depression states of patients before care and 24 weeks after the initiation of care. This scale consists of 14 items, with 7 items related to anxiety (HADS-A) and 7 items related to depression (HADS-D). Each item is scored on a scale from 0 to 3, resulting in a total score range of 0 to 21 for each subscale. Scores below 8 indicate no disorder, while scores above 10 indicate a psychiatric disorder. The Cronbach’s α coefficients for the Chinese versions of HADS-A and HADS-D are 0.855 and 0.879, respectively (17).

##### 2.4.2.3 Pittsburgh sleep quality index

The PSQI was used to assess the sleep quality of patients before care and at 6, 12, 18, and 24 weeks after the initiation of care. This scale includes seven components: subjective sleep quality, sleep latency, sleep duration, habitual sleep efficiency, sleep disturbances, use of sleeping medication, and daytime dysfunction. Each component is scored on a scale from 0 to 3, resulting in a total score range of 0 to 21. Higher scores indicate poorer sleep quality. The Cronbach’s α coefficient for the Chinese version of the PSQI is 0.82 (18).

##### 2.4.2.4) End-of-life chemotherapy

Data were collected on whether patients received chemotherapy within the last 2 weeks, 4 weeks, and 6 weeks of life.

### 2.5 Statistical analysis

Statistical analysis was performed using SPSS software (version 29.0). Continuous variables were expressed as mean ± standard deviation (M ± SD), and categorical variables as frequencies and percentages. Between-group comparisons at each time point were conducted using independent samples t-tests or χ² tests, as appropriate.

Given the repeated measurements at baseline, 6, 12, 18, and 24 weeks, a longitudinal analytical perspective was applied to evaluate trends over time. Comparisons across time points were used to assess changes in central tendencies and outcome trajectories. A p-value < 0.05 was considered statistically significant. For longitudinal assessments, cases with missing data at specific follow-up time points were handled using a complete-case analysis approach. Only available data at each time point were included in the respective analyses, and no imputation methods were applied due to the retrospective nature of the study.

## 3 Results

### 3.1 General information

There were no significant differences observed in the demographic characteristics between the two groups across all evaluated parameters (**Table 1**). There were no significant differences in age, BMI, marital status, education level, payment method, and family monthly income (all P > 0.05) between the groups. These results indicate that the demographic characteristics were evenly distributed between the two groups.

**Table 1:** Comparison of demographic characteristics between the two groups.

Parameter	Usual care group (n=112)	EIPC group (n=88)	t/χ^2^	P
Age (years)	56.43 ± 11.51	54.67 ± 10.84	1.102	0.272
BMI (kg/m^2^)	26.55 ± 3.24	26.81 ± 3.46	0.541	0.589
Marital status [n]			0.269	0.966
Single	19 (16.96%)	13 (14.77%)		
Married	76 (67.86%)	62 (70.45%)		
Divorced	11 (9.82%)	9 (10.23%)		
Widowed	6 (5.36%)	4 (4.55%)		
Education level [n]			0.063	0.969
High school or less	58 (51.79%)	44 (50.00%)		
College	32 (28.57%)	26 (29.55%)		
Graduate school	22 (19.64%)	18 (20.45%)		
Payment method [n]			0.511	0.775
National health insurance	91 (81.25%)	70 (79.55%)		
Private insurance	17 (15.18%)	13 (14.77%)		
Self-pay	4 (3.57%)	5 (5.68%)		
Monthly household income [n]			0.249	0.883
<3000 yuan	37 (33.04%)	28 (31.82%)		
3000~6000 yuan	54 (48.21%)	41 (46.59%)		
>6000 yuan	21 (18.75%)	19 (21.59%)		

EIPC, Early Integrated Palliative Care; BMI, Body Mass Index.

In the comparison of clinical characteristics between the two groups, there were no significant differences in the time of advanced breast cancer diagnosis (P = 0.210), breast cancer molecular subtypes (P = 0.963), number of distant metastatic lesions (P = 0.842), brain metastasis status (P = 0.852), and ECOG performance status scores (P = 0.849; **Table 2**). These results indicate that the clinical characteristics were evenly distributed among the patients included in this study.

**Table 2:** Comparison of clinical features between the two groups.

Parameter	Usual care group (n=112)	EIPC group (n=88)	t/χ^2^	P
Time since diagnosis of advanced breast cancer (months)	23.08 ± 7.46	21.83 ± 6.27	1.257	0.210
Breast cancer molecular subtype [n]			0.075	0.963
HR+/HER2-	68 (60.71%)	54 (61.36%)		
HER2+	22 (19.64%)	18 (20.45%)		
Triple negative	22 (19.64%)	16 (18.18%)		
Number of distant metastatic sites [n]			0.343	0.842
1	45 (40.18%)	38 (43.18%)		
2	40 (35.71%)	28 (31.82%)		
≥3	27 (24.11%)	22 (25.00%)		
Brain metastasis [n]	15 (13.39%)	11 (12.50%)	0.035	0.852
ECOG PS [n]			0.326	0.849
0	35 (31.25%)	30 (34.09%)		
1	58 (51.79%)	42 (47.73%)		
2	19 (16.96%)	16 (18.18%)		

EIPC, Early Integrated Palliative Care; HR, Hormone Receptor; HER2, Human Epidermal Growth Factor Receptor 2; ECOG PS, Eastern Cooperative Oncology Group Performance Status.

### 3.2 Symptom burden

**Figure 1:**
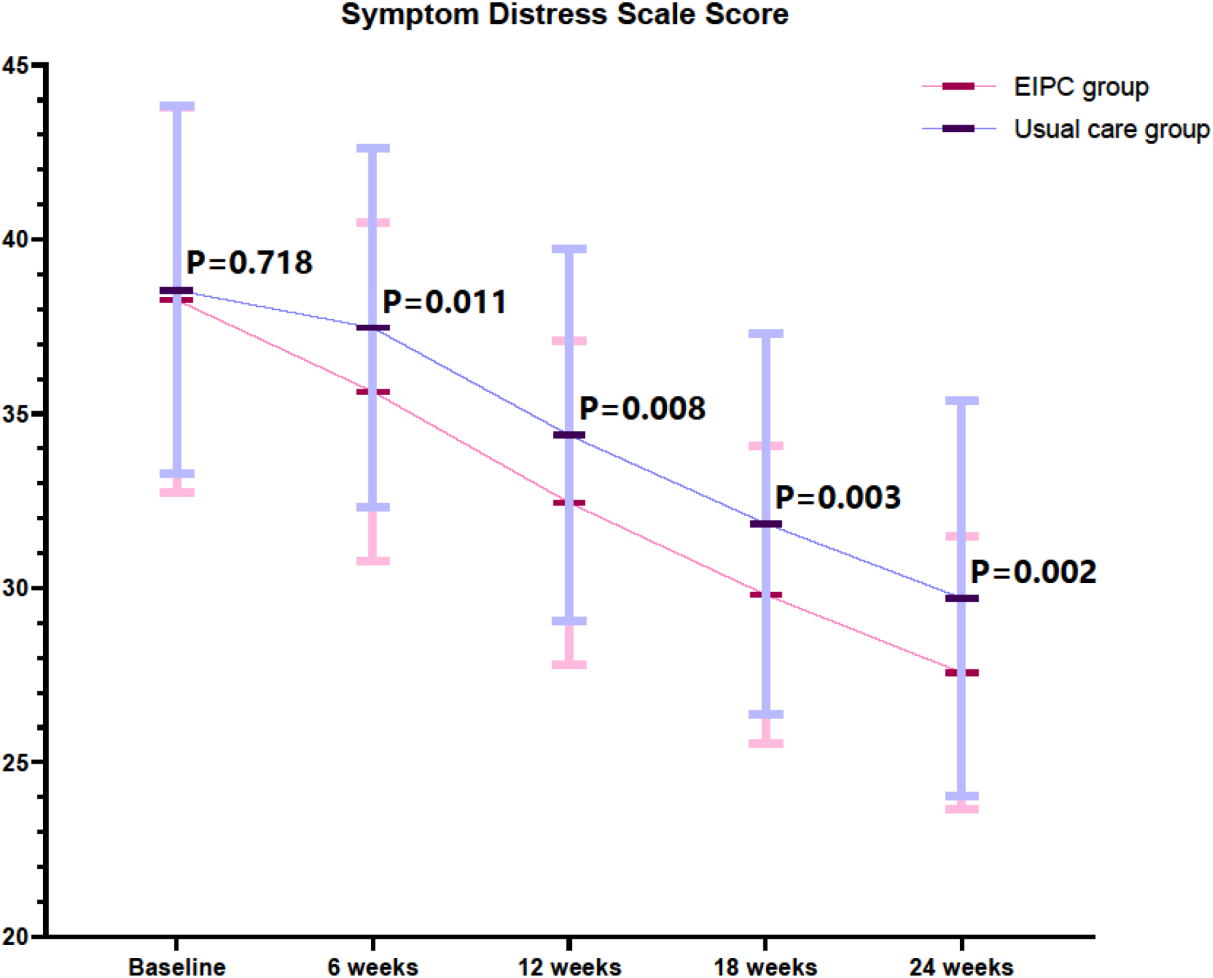
Comparison of SDS between two groups (points). SDS, Symptom Distress Scale; EIPC, Early Integrated Palliative Care.

There was no significant difference in SDS scores between the two groups at baseline (P = 0.718; **Figure 1**). Starting from week 6, the SDS scores in the EIPC group were significantly lower than those in the conventional care group, and this difference persisted until the end of the 24-week observation period (6 weeks: P = 0.011; 12 weeks: P = 0.008; 18 weeks: P = 0.003; 24 weeks: P = 0.002). This indicates that the EIPC intervention may be more effective in reducing patients’ symptom distress.

### 3.3 Quality of life

At baseline, there were no significant differences between the two groups in any dimension of FACT-B (all P > 0.05; **Table 3**). At 24 weeks, the EIPC group showed significantly higher scores than the conventional care group in PWB (P = 0.036), SWB (P = 0.010), EWB (P = 0.003), and FWB (P = 0.008). However, there was no significant difference in BCS between the two groups at 24 weeks (P = 0.819). The significant differences in the other four dimensions suggest that the EIPC intervention may be more effective in improving patients’ overall quality of life.

**Table 3:** Comparison of FACT-B between two groups (points).

Parameter	Usual care group (n=112)	EIPC group (n=88)	t	P
PWB				
Baseline	15.42 ± 3.85	15.74 ± 4.13	0.557	0.578
24 weeks	16.18 ± 4.02	17.32 ± 3.51	2.107	0.036
SWB				
Baseline	14.49 ± 4.35	14.26 ± 4.61	0.368	0.713
24 weeks	17.95 ± 4.28	19.37 ± 3.45	2.591	0.010
EWB				
Baseline	12.35 ± 3.42	12.27 ± 3.55	0.171	0.865
24 weeks	13.08 ± 3.51	14.65 ± 3.72	3.062	0.003
FWB				
Baseline	6.45 ± 1.62	6.62 ± 1.03	0.928	0.355
24 weeks	6.89 ± 1.78	7.54 ± 1.59	2.690	0.008
BCS				
Baseline	19.87 ± 5.42	19.72 ± 5.99	0.186	0.853
24 weeks	20.15 ± 5.63	20.34 ± 6.01	0.229	0.819

FACT-B, Functional Assessment of Cancer Therapy-Breast; EIPC, Early Integrated Palliative Care; PWB, Physical Well-Being; SWB, Social/Family Well-Being; EWB, Emotional Well-Being; FWB, Functional Well-Being; BCS, Breast Cancer Subscale.

### 3.4 Psychological and emotional status

In the comparison of HHI dimension scores between the two groups, there were no significant differences in any dimension at baseline (all P > 0.05; **Table 4**). At 24 weeks, the EIPC group showed significantly higher scores than the conventional care group in having a positive attitude towards reality and the future (P = 0.004), taking positive actions (P = 0.003), and maintaining intimate relationships with others (P = 0.039). These results suggest that the EIPC intervention helps to improve patients’ positive attitudes towards their present and future lives, promote active coping behaviors, and enhance their intimate relationships with others.

**Table 4:** Comparison of HHI between two groups (points).

Parameter	Usual care group (n=112)	EIPC group (n=88)	t	P
A positive attitude toward reality and the future				
Baseline	10.36 ± 1.86	10.48 ± 1.61	0.469	0.640
24 weeks	12.44 ± 1.93	13.06 ± 0.98	2.958	0.004
Taking positive action				
Baseline	10.92 ± 1.79	11.27 ± 1.64	1.422	0.157
24 weeks	12.23 ± 1.81	12.89 ± 1.28	3.020	0.003
Maintaining close relationships with others				
Baseline	11.06 ± 1.82	11.15 ± 1.72	0.344	0.731
24 weeks	11.12 ± 1.85	11.67 ± 1.84	2.081	0.039

HHI, Herth Hope Index; EIPC, Early Integrated Palliative Care.

**Figure 2:**
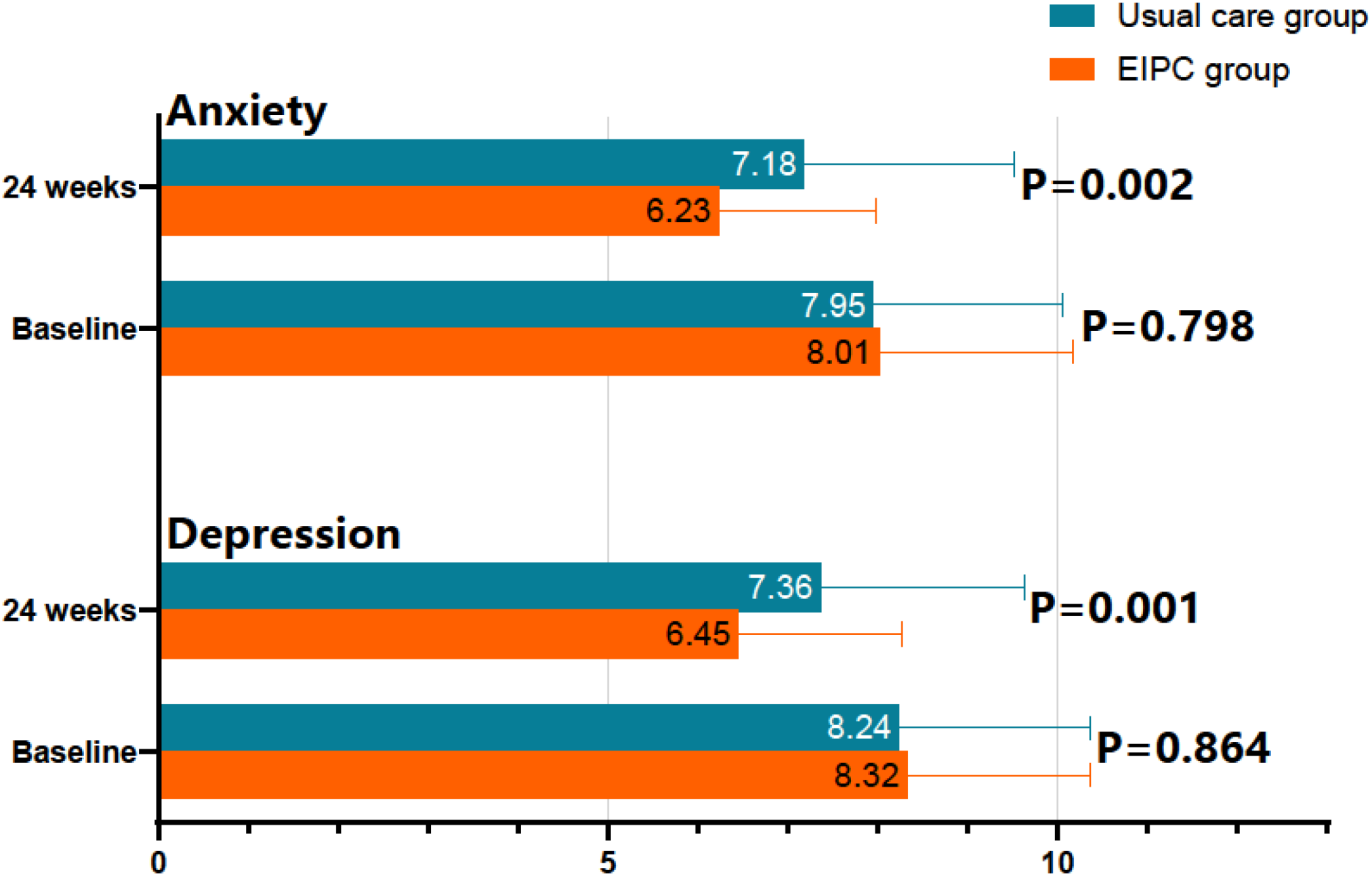
Comparison of HADS between two groups (points). HASD, Hospital Anxiety and Depression Scale; EIPC, Early Integrated Palliative Care.

The HADS scores between the two groups showed no significant differences at baseline, either in the depression dimension (P = 0.798) or the anxiety dimension (P = 0.864; **Figure 2**). At 24 weeks, the EIPC group exhibited significantly lower scores than the conventional care group in both the depression (P = 0.002) and anxiety (P = 0.001) dimensions. These results suggest that the EIPC intervention demonstrates a clear advantage in reducing levels of depression and anxiety.

### 3.5 Sleep quality

There were no significant differences in PSQI scores between the two groups at baseline (P = 0.595; **Table 5**). Starting from week 6, the EIPC group showed significantly lower PSQI scores compared to the conventional care group, and this difference persisted until the end of the 24-week observation period (6 weeks: P = 0.020; 12 weeks: P = 0.005; 18 weeks: P = 0.003; 24 weeks: P = 0.002). At each assessment time point, the sleep quality scores of the EIPC group were significantly lower than those of the conventional care group, indicating that the EIPC intervention is more effective in improving patients’ sleep quality.

**Table 5:** Comparison of PSQI between two groups (points).

Parameter	Usual care group (n=112)	EIPC group (n=88)	t	P
Baseline	11.27 ± 2.85	11.48 ± 2.76	0.533	0.595
6 weeks	10.69 ± 2.83	9.81 ± 2.42	2.342	0.020
12 weeks	9.72 ± 2.96	8.66 ± 2.34	2.828	0.005
18 weeks	9.04 ± 2.97	7.93 ± 2.15	3.049	0.003
24 weeks	8.19 ± 2.38	7.21 ± 2.04	3.081	0.002

PSQI, Pittsburgh Sleep Quality Index; EIPC, Early Integrated Palliative Care.

### 3.6 End-of-life chemotherapy

In the comparison of the rates of terminal chemotherapy between the two groups, there was no significant difference in the last 2 weeks of life (P = 0.387; **Table 6**). In the last 4 weeks of life, although the conventional care group had a higher chemotherapy rate than the EIPC group, this difference did not reach statistical significance (P = 0.087). In the last 6 weeks of life, the chemotherapy rate in the EIPC group was significantly lower than that in the conventional care group (P = 0.003). This suggests that EIPC may help reduce the need or willingness of patients to undergo chemotherapy during their final stages of life. These results highlight the potential value of EIPC in providing comprehensive support and optimizing end-of-life care, particularly in reducing unnecessary invasive treatments.

**Table 6:** Comparison of the end-of-life chemotherapy rate between the two groups [n(15)].

Parameter	Usual care group (n=112)	EIPC group (n=88)	χ^2^	P
In the last 6 weeks of life	45 (40.18%)	18 (20.45%)	8.885	0.003
In the last 4 weeks of life	26 (23.21%)	12 (13.64%)	2.937	0.087
In the last 2 weeks of life	10 (8.93%)	5 (5.68%)	0.749	0.387

EIPC, Early Integrated Palliative Care.

## 4 Discussion

This retrospective cohort study provides compelling evidence supporting the multifaceted benefits of a structured EIPC model for patients with advanced ABC. Compared to usual oncologic care, EIPC intervention was associated with significant and sustained improvements in a range of patient-centered outcomes, including physical symptom burden, multidimensional quality of life, psychological and emotional well-being, sleep quality, and key end-of-life care decisions. These findings underscore the critical value of embedding a patient-centered, holistic care model early in the disease continuum rather than relegating palliative interventions to the final stages.

The observed reduction in overall symptom distress in the EIPC group aligns with the core mission of palliative care. It corroborates key trial results from other malignancies such as lung and pancreatic cancer (19, 20). Proactive, standardized assessments using tools like the SDS may enable multidisciplinary teams to identify and address symptoms such as pain, fatigue, and nausea before they reach severe levels. This targeted, preemptive management involving expert pharmacological and non-pharmacological strategies stands in stark contrast to the reactive, often oncologist-led approaches in routine care, which may prioritize treatment-related toxicities over the broader symptom cluster (20). The gradual divergence in SDS scores over 24 weeks suggests that the benefits of EIPC are not only immediate but also cumulative, potentially due to sustained support and education enhancing patient self-efficacy in symptom reporting and management (21). Our findings align with previous research showing that early palliative care integration improved symptom trajectories in non-small cell lung cancer patients (22).

Alongside symptom relief, the EIPC group showed improvements across multiple domains of cancer treatment, including breast function assessment. BCS often captures issues directly related to the disease and its treatment, such as satisfaction with body image and sexual function, which may be less amenable to supportive care interventions in the short term compared to broader aspects of well-being (23). Improvements in the other four domains highlight the holistic effect of EIPC. Enhanced physical health may directly stem from better symptom control. Improvements in social/family well-being can be attributed to the involvement of social workers, family meetings, and support groups facilitated by the EIPC team, which can alleviate caregiver burden and improve communication, a finding echoed in family-centered palliative care studies (24). Improvements in emotional and functional health represent a powerful synergy. By alleviating physical suffering and providing structured psychological support, patients may experience less emotional exhaustion and regain a greater sense of purpose and ability to engage in meaningful activities, thereby improving functional status (25). This multidimensional quality of life improvement exceeds the typically more modest improvements reported in some earlier ABC studies, possibly due to the comprehensiveness and early timing of the intervention assessed here (26). Notably, the absence of a statistically significant difference in the breast cancer-specific subscale (BCS) warrants further consideration. This may be attributed to the nature of BCS items, which primarily reflect disease- and treatment-specific concerns such as body image and sexual functioning. These aspects are often less responsive to palliative care interventions over a relatively short follow-up period and may require longer-term or specialized interventions to demonstrate measurable change.

The observed psychological benefits were not limited to reducing negative impacts but also included actively cultivating positive psychological resources, namely hope. The reduction in anxiety and depression HADS scores in the EIPC group is consistent with numerous studies demonstrating the effectiveness of integrated palliative care and psychosocial interventions in improving mood disorders (27). The novel and significant finding of enhanced hope, as measured by the Herth Hope Index, is particularly noteworthy. The enhancement of hope, characterized by a more positive attitude towards the future, more active engagement, and stronger interpersonal relationships, underscores the transformative potential of early palliative care in reshaping patients’ perspectives on living (28). Our findings suggest that EIPC fosters a shift in hope—from a narrow focus on cure to a broader, more attainable hope for symptom relief, meaningful time with loved ones, achieving personal goals, and spiritual peace. The structured communication and ACP processes within EIPC may help patients and families cultivate realistic expectations, thereby reducing anxiety associated with uncertainty and fostering a hopeful yet grounded outlook (29). This aligns with contemporary theories of hope in serious illness and distinguishes EIPC from interventions that solely focus on psychopathology (30).

The improvement in sleep quality in the EIPC group adds another critical dimension to its benefits. Sleep disorders in ABC are multifactorial, caused by physical symptoms (pain, dyspnea), psychological distress (anxiety, depression), and treatment side effects (31). The EIPC model addresses these root causes simultaneously, providing a plausible mechanism for the observed improvements. Better pain and symptom management directly alleviates sleep disturbances (32). Psychological interventions help reduce anxiety and depression, mitigating cognitive arousal and nighttime worries. This outcome is supported by an increasing body of literature linking palliative care interventions with improved sleep outcomes, emphasizing a previously underappreciated but crucial aspect of patient comfort that EIPC can positively influence.

Another notable observation in our study was the reduced chemotherapy rate in the EIPC group during the last six weeks of life. The difference at six weeks suggests that EIPC enables an earlier and more deliberate transition in care goals. Through ongoing, compassionate conversations about prognosis, values, and the potential burdens versus benefits of continued cancer-directed therapy, early palliative care may facilitate more nuanced and realistic discussions about the benefits and burdens of active treatment near the end of life (33). This finding strongly supports the work and extends it to the cancer population, indicating that early integration of palliative care can alter the aggressiveness of end-of-life care, which is associated with better quality of death and reduced bereavement for caregivers (34). The observed reduction in chemotherapy use during the final weeks of life should be interpreted within the framework of integrated palliative care rather than as a withdrawal of active treatment. EIPC supports shared decision-making by continuously evaluating the balance between treatment benefits and burdens. This approach enables a transition from disease-centered to goal-concordant care when appropriate, thereby reducing potentially non-beneficial aggressive interventions at the end of life. This finding aligns with the concept of simultaneous care, where palliative and oncologic treatments coexist, ensuring that therapeutic decisions remain individualized and patient-centered.

Despite these promising findings, several limitations warrant careful consideration. The retrospective and non-randomized design introduces the possibility of selection bias and unmeasured confounders. Although baseline demographic and clinical characteristics were balanced, unobserved variables such as physician referral patterns, inherent patient resilience, or social support networks could influence EIPC uptake and outcomes. As a single-center study, it may limit the generalizability of the findings to other institutions with varying palliative care resources, cultural backgrounds, or healthcare systems. Additionally, cultural factors and healthcare system characteristics in China, including family-centered decision-making, resource availability, and varying levels of palliative care integration, may influence the implementation and outcomes of EIPC. These contextual factors should be considered when generalizing the findings to other regions. Future studies should explore the adaptability of EIPC models across diverse healthcare systems and cultural environments. Recent literature also highlights the importance of integrating multidisciplinary and culturally sensitive approaches in breast cancer management (35). The 24-week observation period, while substantial, may not capture the full longitudinal trajectory of benefits, particularly in long-term survival, late-onset symptoms, or stability of hope and ACP decisions. Future research should address these limitations through prospective, multicenter randomized controlled trials with longer follow-up periods. Studies should also explore specific components of EIPC, determine optimal timing and intensity of integration, and examine its effectiveness across different healthcare settings and cultural populations.

## 5 Conclusion

This study further demonstrates that early integrated palliative care is a powerful, multifaceted intervention for patients with advanced cancer. By proactively addressing symptoms, providing structured psychosocial support, facilitating clear communication about goals, and coordinating care, EIPC significantly reduces suffering, improves quality of life and hope, enhances sleep, and promotes more rational, patient-centered decision-making at the end of life.

## Data Availability

The raw data supporting the conclusions of this article will be made available by the authors, without undue reservation.
